# Increased left ventricular torsion in hypertrophic cardiomyopathy mutation carriers with normal wall thickness

**DOI:** 10.1186/1532-429X-13-3

**Published:** 2011-01-10

**Authors:** Iris K Rüssel, Wessel P Brouwer, Tjeerd Germans, Paul Knaapen, Tim J Marcus, Jolanda van der Velden, Marco JW Götte, Albert C van Rossum

**Affiliations:** 1 Department of Clinical Physics, Hagaziekenhuis, The Hague, The Netherlands; 2 Department of cardiology, Hagaziekenhuis, The Hague, The Netherlands; 3 Department of Cardiology, VU University Medical Center, Amsterdam, The Netherlands; 4 Department of Physics and Medical Technology, VU University Medical Center, Amsterdam, The Netherlands; 5 Department of Physiology, VU University Medical Center, Amsterdam, The Netherlands; 6 Interuniversity Cardiology Institute of the Netherlands (ICIN), Utrecht, The Netherlands

## Abstract

**Background:**

Increased left ventricular (LV) torsion has been observed in patients with manifest familial hypertrophic cardiomyopathy (HCM), and is thought to be caused by subendocardial dysfunction. We hypothesize that increased LV torsion is already present in healthy mutation carriers with normal wall thickness.

**Methods:**

Seventeen carriers with an LV wall thickness <10 mm, and seventeen age and gender matched controls had cardiovascular magnetic resonance (CMR) cine imaging and tissue tagging. LV volumes and mass were calculated from the cine images. LV torsion, torsion rate, endocardial circumferential strain and torsion-to-endocardial-circumferential-shortening (TECS) ratio, which reflects the transmural distribution in contractile function, were determined using tissue tagging.

**Results:**

LV volumes, mass and circumferential strain were comparable between groups, whereas LV ejection fraction, torsion and TECS-ratio were increased in carriers compared to controls (63 ± 3% vs. 60 ± 3%, p = 0.04, 10.1 ± 2.5° vs. 7.7 ± 1.2°, p = 0.001, and 0.52 ± 0.14°/% vs. 0.42 ± 0.10°/%, p = 0.02, respectively).

**Conclusions:**

Carriers with normal wall thickness display increased LV torsion and TECS-ratio with respect to controls, which might be due to subendocardial myocardial dysfunction. As similar abnormalities are observed in patients with manifest HCM, the changes in healthy carriers may be target for clinical intervention to delay or prevent the onset of hypertrophy.

## Introduction

Hypertrophic cardiomyopathy (HCM) is characterized by asymmetrical (septal) hypertrophy in the absence of increased external load and is caused by mutations in mainly sarcomeric genes that have an autosomal-dominant pattern of inheritance [1,2]. Several echocardiographic and magnetic resonance studies demonstrated abnormal left ventricular (LV) systolic and diastolic function in patients with manifest HCM [3-5]. As the genetic defect is present from birth, family members of patients with manifest HCM are at risk to develop HCM. However, at present, little is known about changes in cardiac function which precede cardiac hypertrophy. Identification of abnormalities in cardiac function before the onset of hypertrophy may provide a basis for clinical intervention to delay or prevent the progression to manifest HCM in mutation carriers.

In the healthy heart, myocardial deformation is predominantly based on contraction of helically oriented bundles of myofibers in the endo- and epicardium [6]. A parameter which directly relates to the functional status of these bundles of myofibers is LV torsion [7]. Torsion is described as the overall twisting or 'wringing' motion of the heart caused by an opposite rotation of the base and apex. Torsional deformation is important during LV contraction, since it distributes strain homogeneously over the LV wall [8]. In the absence of torsion, subendocardial fibers would shorten to a greater extent than subepicardial fibers, leading to non-uniform transmural fiber shortening. Mathematical models [8] have shown that a well balanced interplay between torsion and ejection volume is necessary to diminish differences between subendo- and subepicardial fiber contraction. Since LV ejection is directly dependent on subendocardial circumferential strain (area reduction in the short axis directly implies shortening of the circumference), the constant ratios of torsion to subendocardial circumferential strain (so called torsion-to-shortening ratio (TSR) [9]), detected in humans and animals with structural normal hearts, confirm the interplay between LV ejection and torsion in vivo [9,10]. Changes in this ratio indicate transmural differences in fiber shortening.

Altered torsion is found to be a sensitive marker for both systolic and diastolic dysfunction [11-18]. Elevated torsion and/or TSR-ratios have been detected almost exclusively in patients with LV hypertrophy caused by increased hemodynamic loading conditions (i.e. aortic valve stenosis [11,17] or hypertension [19]), and in HCM patients [20]. However, elderly with normal LV wall dimensions also show increased left ventricular torsion, indicating that age-related myocardial alterations contribute to changes in the pattern of contraction in this population [12].

Histological studies [21-25] revealed a variety of structural myocardial abnormalities in manifest HCM (i.e. myocardial disarray, thickening of the intima layer of small subendocardial arterioles and areas of fibrosis). As myocardial architecture might already be altered in pre-hypertrophied mutation carriers, we hypothesize that torsion, and hence, the ratio between torsion and subendocardial strain, is altered in HCM mutation carriers with normal wall thickness. In the present study LV torsion was evaluated in mutation carriers and an age and gender matched control population using cardiovascular magnetic resonance (CMR).

## Methods

### Subjects

Seventeen HCM mutation carriers and seventeen age and gender matched controls without medical history (e.g. no evidence of other systemic or cardiac disease associated with LV hypertrophy) were included in the study. Families of control patients were deprived of sudden cardiac death and cardiomyopathies. Carriers were first-degree relatives of HCM index patients and recruited after genetic testing at the cardiogenetic outpatients' clinic. They had either a 2373insG mutation in the gene encoding for cardiac myosin binding protein C (MYBPC3) [26], or a Glu62Gln missense mutation in the gene encoding for alpha-tropomyosin (TPM1) [27], as described previously [28]. All carriers had a LV wall-thickness <10 mm and showed no signs of LV outflow tract obstruction, as measured by routine echocardiography within one year prior to inclusion. In all participants, a standard physical examination was executed directly before CMR acquisition. The study was approved by the institutional medical ethics committee and conducted according to the declaration of Helsinki. Written informed consent was obtained from all participants before entering the study.

### Cardiac magnetic resonance imaging

All subjects were imaged on a 1.5 T whole body scanner (Magnetom Sonata, Siemens, Erlangen, Germany), using a six-channel phased-array body coil. All cine studies were performed in a single breath hold during mild expiration.

**Figure 1:**
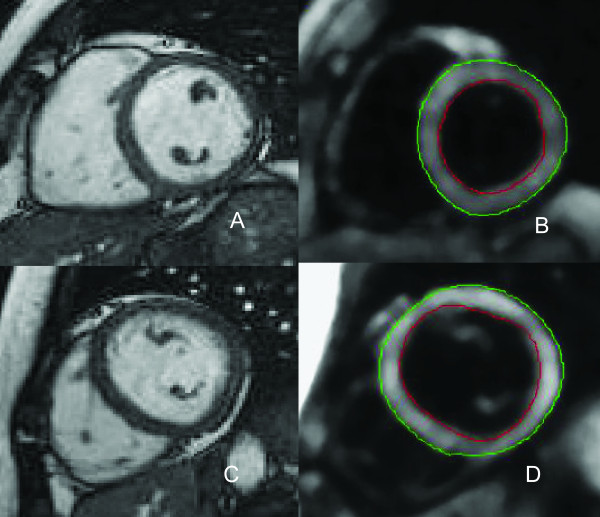
**CMR techniques used for morphological and functional assessment**. A: End-diastolic basal short-axis SSFP image of a healthy control, used for quantification of LV volumes and mass. B: LV basal short-axis end-diastolic HARM-image of the same patient as (A) with manually drawn epicardial (green) and endocardial (red) contours. By using Harmonic phase (HARP) analysis, myocardial deformation can be monitored throughout the cardiac cycle, allowing determination of left ventricular torsion and subendocardial circumferential strain (inner 50% of the LV wall) throughout the cardiac cycle. C: End-diastolic short axis SSFP image of a HCM mutation carrier, showing normal cardiac morphology and dimensions. D: LV short axis HARM-image of the same carrier as (C) acquired at end-systole.

Retro-triggered, balanced steady-state free precession (SSFP) short-axis cine imaging with full coverage of the LV was acquired for quantification of LV volumes, mass and ejection fraction (EF) (Figure 1). Image parameters were: slice thickness 5 mm, slice gap 5 mm, temporal resolution <50 ms, 20 phases per cardiac cycle, echo time 1.54 ms, repetition time 3.2 ms, flip angle 60 degrees and a typical image resolution of 1.3*1.6 mm. For analysis of left atrial (LA) volume, the same acquisition was performed in a transversal orientation, planned on an end-diastolic 2-chamber view at the level of the lower leading edge of the mitral valve annulus as described previously [29]. The LA was subsequently fully covered by a stack of transversely oriented slices.

A high temporal resolution (14 ms) 3-chamber cine image was performed to determine the opening and closing time of the valves.

In addition, in 15 of the control subjects and in 13 of the HCM mutation carriers, velocity encoded cine CMR was applied for quantification of mitral valve inflow velocities in order to obtain E/A ratios as reflection of diastolic function. Flow measurements were performed in a retrospective fast low angle shot (FLASH) phase-contrast technique with a velocity sensitivity set at 100 cm s^-1 ^[30].

Myocardial tissue tagging (Figure 1) was performed at three short-axis slices, located at ¼ (base), 1/2 (mid), and ¾ (apex) of the length between the mitral valve and the apex, planned on an end-systolic four-chamber image. Sinusoidal, complementary tagged (CSPAMM) images were acquired with a retro-triggered SSFP sequence using a multiple brief expiration breath hold scheme [31]. Temporal resolution was 15 ms. The imaging parameters were as follows: field of view: 300 × 300 mm^2^, flip-angle: 20°, repetition time: 3.6 ms, echo time: 1.8 ms, receiver bandwidth: 850 Hz/pixel, matrix size: 256 × 78, slice thickness: 6 mm, tag-line distance: 7 mm. Acquisition time per slice was approximately 3-4 minutes. After tagging images were derived, we administered 0.2 mmol/kg Gadolinium-DTPA (Magnevist, Schering^©^) solely to the HCM mutation carriers, in order to obtain Late Gadolinium Enhancement (LGE) images for the detection of intramyocardial fibrosis.

### Post-processing

LV and LA volumes, mass and blood-flow velocities were analyzed offline, using dedicated software packages (Mass and Flow, Medis, Leiden, the Netherlands). From these, volumes, ejection fraction (EF), stroke volume (SV) and E/A ratio were derived.

From the tagging images, LV torsion was calculated as the circumferential-longitudinal shear angle using Harmonic Phase displacement tracking as described before [32,33]. For this, endo- and epicardial contours were drawn on the harmonic magnitude (HARM) images (Figure 1) [34]. Since this method includes the radius and the length of the ventricle, torsional values are comparable between hearts of different sizes. Torsion was calculated between the basal and apical slices, between the basal and mid slices and between the mid and apical slices.

From the torsion curves, peak torsion was derived. Peak rate of systolic and diastolic torsion was determined by calculating the time derivative of the torsion curves, with a temporal resolution of three phases (45 ms). The peak rates of systolic and diastolic torsion were corrected for peak torsion, since this parameter has been shown to increase in proportion to the value of peak torsion during exercise [35].

Furthermore, Lagrangian circumferential strain was calculated from the tagging images using the Harmonic Phase method as described previously [36]. Strains were assessed for the inner (subendocardial) layer of the myocardium, encompassing 50% of the LV wall. Therefore, the epicardial contour (green line in Figure 1) was automatically shifted towards the centre of the wall. Peak values were determined from the strain curves.

Besides, the ratio of peak LV torsion to peak endocardial circumferential shortening (TECS-ratio) was calculated as an alternative to TSR. We must note that TECS and TSR are not comparable in absolute terms, since the calculation of subendocardial circumferential strain is based upon different definitions (TSR see [10-12,17]).

### Statistical analysis

All data are presented as mean ± SD. LA and LV volumes, LV mass, function, torsion, torsion rates and circumferential strain were compared between the carrier and the control group using Student's T-test or Mann-Whitney U test, when appropriate. Torsion was calculated on different longitudinal levels (base-mid, mid-apex) and compared within patients using paired Student's T-test. Interobserver variability in peak LV torsion was assessed by redrawing of myocardial contours in 5 randomly selected healthy subjects and carriers and recalculating peak torsion by two independent observers (WPB and IKR). Bland-Altman analysis and intraclass correlation (ICC) were assessed to determine agreement. Two-sided p-values < 0.05 were considered as statistically significant.

## Results

Subject characteristics are presented in Table 1. No differences in baseline characteristics were observed between the carriers and the control group. In total, mutation carriers from 10 different families were included. The majority of carriers (13/17) had a MYBPC3 mutation, and four had a mutation in the TPM1 gene.

**Table 1:** Baseline characteristics.

	Carriers (n = 17)	Controls (n = 17)	p-value
Age (years)	40 ± 12	38 ± 13	n.s.

Gender (male/female)	5/12	8/9	n.s.

Systolic blood pressure (mmHg)	115 ± 12	118 ± 7	n.s.

Diastolic blood pressure (mmHg)	66 ± 9	69 ± 7	n.s.

Heart rate (bpm)	63 ± 9	68 ± 8	n.s.

Body surface area (m^2^)	1.90 ± 0.19	1.90 ± 0.21	n.s.

In Table 2, volumes and function analysis can be found. The EF was slightly but significantly higher in carriers (p = 0.04). The LV mass and volumes were similar in both groups. Furthermore, indices of diastolic function (E/A ratio, LA volumes and isovolumetric relaxation time (IVRT)) showed no significant differences between carriers and controls.

**Table 2:** LV and LA volumes and function.

	Carriers (n = 17)	Controls (n = 17)	p-value
LV mass (g)	104 ± 26	104 ± 30	n.s.

LV mass/EDV ratio (g/ml)	0.59 ± 0.13	0.57 ± 0.08	n.s.

LV mass/BSA (g/m^2^)	54.6 ± 11.4	54.4 ± 11.4	n.s.

LVEDV (ml)	177 ± 29	180 ± 35	n.s.

LVESV (ml)	67 ± 12	72 ± 16	n.s.

LV Stroke volume (ml)	111 ± 19	108 ± 20	n.s.

LV Ejection fraction (%)	63 ± 3	60 ± 3	0.04

LAEDV (ml)	49 ± 16	40 ± 9	n.s.

LAESV (ml)	111 ± 30	101 ± 20	n.s.

IVRT (ms)	96 ± 14	92 ± 18	n.s.

E/A ratio	1.62 ± 0.70	1.64 ± 0.61	n.s.

* EDV: end-diastolic volume, ESV: end-systolic volume. Isovolumetric relaxation time (IVRT); results are mean ± SD; n.s. = non significant

**Figure 2:**
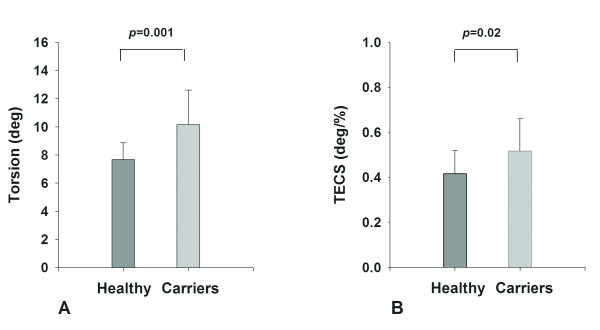
**Error bar plots of Peak LV Torsion (A) and TECS-ratio (B) (torsion to endocardial circumferential shortening) in the control and carrier group**. The difference between both groups is significant.

A significant increase in peak base-apex torsion was found in HCM mutation carriers with respect to controls (p = 0.001) (Table 3, Figure 2), while peak circumferential strain was similar. Hence, TECS-ratio was significantly increased in the carrier group (Figure 2).

**Table 3:** Torsion analysis.

	Carriers (n = 17)	Controls (n = 17)	p-value
Peak torsion (°)	10.1 ± 2.5	7.7 ± 1.2	0.001

Peak systolic torsion rate (°/s)	53 ± 15	39 ± 5	0.001

Peak diastolic torsion rate (°/s)	-71 ± 17	-54 ± 13	0.003

Peak systolic torsion rate/Peak torsion (s^-1^)	5.2 ± 1.2	5.2 ± 8.9	n.s.

Peak diastolic torsion rate/Peak torsion (s^-1^)	-7.2 ± 1.7	-7.2 ± 1.8	n.s.

Peak endocardial circumferential strain (%)	19.9 ± 2.8	18.9 ± 2.6	n.s.

TECS-ratio (°/%)	0.52 ± 0.14	0.42 ± 0.10	0.02

Results are mean ± SD; n.s. = non significant

**Figure 3:**
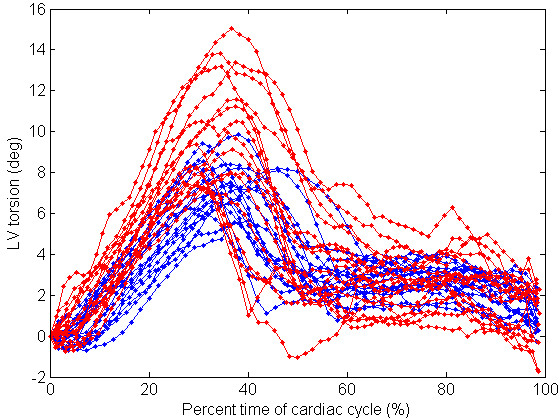
**Individual, interpolated LV torsion curves for carriers (blue) and controls (red)**. Individual curves were interpolated to 100 points over their cardiac cycle.

No significant differences could be detected in peak systolic and diastolic torsion rate, after correction for peak torsion. This is also shown in Figure 3, which visually outlines that increased torsion is accompanied with a similar increment of (uncorrected) torsion rates. There was no significant difference between torsion measured at different levels (base-mid, mid-apex) of the LV (data not shown).

A total of 4 mutation carriers showed small areas of focal intramyocardial contrast enhancement. In order to rule out a potential influence of fibrosis on torsional behaviour and TECS measurements, we compared the values between LGE positive and LGE negative HCM mutation carriers. Results showed non-significant differences between both groups: 9.3 ± 1.85° vs 10.4 ± 2.63°, p = ns for torsion, and 0.54 ± 0.08°/% vs 0.51 ± 0.16°/%, p = ns for TECS respectively.

**Figure 4:**
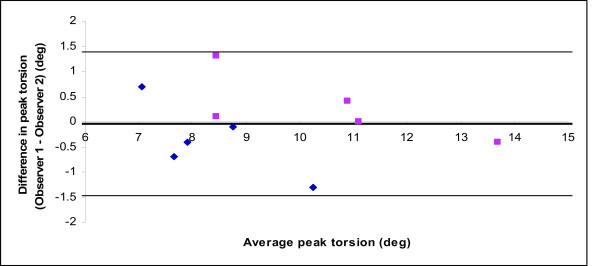
**Bland Altman plot of peak torsion values as analyzed by two independent observers**. Agreement is similar in healthy subjects (blue lozenges) and carriers (magenta squares).

Inter-observer agreement for peak torsion was high, with an ICC of 0.94 (95% confidence interval: 0.79-0.99), and limits of agreement are -0.1 ± 16.4% (Figure 4). It should be noted that inter-observer agreement is only dependent on drawing of contours, as further calculation of torsion and its peak value is observer-independent.

## Discussion

Our study demonstrates that HCM mutation carriers have increased LV torsion in the absence of left ventricular hypertrophy. Most studies evaluating LV rotation and/or torsion were performed in patients with underlying conditions causing overt (concentric) hypertrophy, like hypertension or aortic valve stenosis [11,13,17,20]. Increased torsion is generally regarded as the result of impaired contraction of predominantly subendocardial myofibers (due to -relative- hypoperfusion of the thickened wall), leading to diminished counteraction of contracting epicardial fibers, which have a longer lever arm [8,17].

Remarkably, non-hypertrophied HCM carriers show similar contraction patterns including a relative increase in LV torsion with respect to subendocardial circumferential contraction (e.g. increased TECS-ratio). It was expected that, due to the elevated ejection fraction in carriers, torsion and subendocardial circumferential strain (which is related to ejection volume) values would show parallel increments, resulting in unaltered TECS-ratio. Apparently, impairment of subendocardial contraction of myofibers changed the balance between torsion and ejection in mutation carriers irrespective of LV wall thickness.

A variety of disease-related morphological and functional alterations could have attributed to this finding. Although currently unknown, changes in transmural distribution and/or angulation of subendocardial myofibers might have influenced regional contraction patterns in carriers. Besides, perturbations of myocardial perfusion cannot be ruled out, since studies in HCM hearts revealed abnormalities in small intramural coronary arteries and subendocardial arterioles [22,23]. A similar observation of increased LV torsion with unaltered circumferential strain in asymptomatic Type I Diabetes Mellitus patients without morphological evidence of cardiac disease [37], contributes to the idea that intrinsic disease-related pathology (e.g. small vessel disease was not ruled out) might be responsible for differences in myocardial deformation. However, future perfusion studies are needed to validate this unconfirmed hypothesis.

Except for myocardial disarray and/or disturbed regional myocardial perfusion as potential explanations for the relatively impaired subendocardial fiber shortening, other factors might be affecting LV torsion in HCM mutation carriers. Interestingly, mutation carriers showed a (slightly) increased ejection fraction (causing the difference in TECS to be less pronounced than the difference in torsion alone (Figure 2)), which is also a common finding in manifest HCM [38].

This seems in line with the detected rises in systolic and diastolic torsional rates [35,39,40] under resting conditions (e.g. normal blood pressure and heart rate), suggesting that the hearts of carriers are in a hypercontractile state at baseline. On the basis of in vitro studies with recombinant mutant sarcomeric proteins and transgenic animal models, it has been proposed that HCM mutations in sarcomeric proteins cause hypercontractility [41]. Hence, the in vivo changes in cardiac performance in mutation carriers found in the present study may be a first indication of altered sarcomeric function, extrapolated to global functional changes of the pre-hypertrophied HCM muscle.

### Limitations and clinical implications

The number of patients enrolled in the study is limited, and therefore, results should be interpreted with care. Nevertheless, the data indicate that significant changes in LV torsion are present in HCM mutation carriers. Besides, not all control subjects were genetically screened for HCM mutations, which theoretically might have biased results, although prevalence is rather low for the general population. In addition, since HCM is associated with an abundance of defects in sarcomeric genes, findings in carriers with only two types of mutations might not be entirely representative for the general HCM population.

Finally, we did not perform LGE imaging in healthy individuals and cannot rule out the presence of fibrosis in these patients. Nevertheless, the probability to encounter areas of focal fibrosis in healthy subjects is very low, and moreover, we demonstrated that the presence of LGE has little significance on torsional behaviour and TECS-values in mutation carriers.

**Figure 5:**
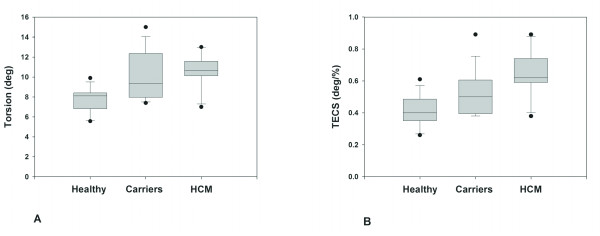
**Box plots displaying the differences in torsion (A) and TECS (B) between healthy controls, carriers and manifest HCM patients**. There is a gradual increase in LV torsion visible from controls towards HCM patients.

To clarify the meaning and potential clinical implications of increased torsional values in HCM carriers, we compared our findings with a group of 10 aged matched patients (44 ± 8 years) with manifest HCM. As Figure 5 outlines, manifest HCM patients have elevated torsion and TECS with respect to controls and carriers, with mean values of 10.6 ± 1.6° and 0.64 ± 0.13°/%, respectively. Carriers have values in between controls and patients with manifest HCM, indicating that the hearts of carriers show deformational alterations preceding the development of hypertrophy. This suggests that the mutation carriers are in a transitional phase towards manifest HCM, although future prospective studies are needed to verify this hypothesis. Early recognition of the abnormality in LV contraction might imply a closer monitoring of these patients at different timeframes. Besides, targeted drug therapy might be applied, aimed at delaying or preventing wall thickening and associated symptoms.

## Conclusion

This study demonstrates that increased torsional deformation already exists in healthy mutation carriers, even in the absence of hypertrophy. Although the exact underlying mechanisms are currently unknown, we speculate that disease related subendocardial dysfunction might be responsible for alterations in torsion.

## Competing interests

The authors declare that they have no competing interests.

## Authors' contributions

IKR and WB: Study design, writing the manuscript, data analysis and interpretation; TG: Study design, revising the manuscript, patient inclusion, acquiring CMR data, coordination of genetic screening; PK: Study design, data interpretation, revising the manuscript; JTM: CMR protocol design, data interpretation, revising the manuscript; JvdV: Study design, data interpretation, revising the manuscript; MJWG: Study design, data interpretation, revising the manuscript; ACvR: CMR protocol design, revising the manuscript.
